# Effects of Culture History and Algal Taxonomy on Bacterial Communities in Long-maintained Filamentous Green Algae

**DOI:** 10.1007/s00248-026-02840-0

**Published:** 2026-07-22

**Authors:** Ethan Wood, Bente Edvardsen, Kari Skjånes, Luka Šupraha

**Affiliations:** 1 Norwegian Institute for Water Research (NIVA), Økernveien 94, Oslo, 0579 Norway; 2 Department of Biosciences, University of Oslo (UIO), Blindernveien 31, Oslo, 0316 Norway; 3 Norwegian Institute of Bioeconomy Research (NIBIO), Høgskoleveien 7, Ås, 1433 Norway

**Keywords:** Phycosphere, Chlorophyta, 16S rRNA metabarcoding, Historical contingency, Culture history, Co-culture, Community coalescence, Host specificity

## Abstract

Algal cultures are widely used to study algae–bacteria interactions, yet the relative roles of algal taxonomy, culturing environment, and historical contingency in shaping bacterial communities of long-maintained monoalgal cultures remain unclear. In a two-stage experimental design, we used 16 S rRNA metabarcoding to first profile the bacterial communities of 14 filamentous freshwater algal strains from three orders (Chaetophorales, Oedogoniales, Zygnematales) maintained in two distinct culture collections, and then, in a second experiment to test community mixing using a co-culture and re-isolation design with two algal strains (*Stigeoclonium* sp. and *Oedogonium* sp.). The 14-strain profiling showed that bacterial communities were broadly similar at higher taxonomic ranks, with many shared orders within Pseudomonadota, whereas composition at the ASV level was largely algal-strain-specific. Ordination of phylogeny-aware UniFrac distances showed that culture collection of origin explained more variation in community composition than algal order, an effect reduced but not eliminated after standardizing the culture medium. The co-culture and re-isolation experiment showed that the two algal strains exchanged many bacterial community members, yet algal strain identity remained detectable after re-isolation. These results support a framework where culture history results in strain-specific ASV-level composition within recurrent higher-rank structure, and where algal-strain-specific effects on bacterial community composition become evident when cultures are exposed to a shared bacterial pool. Co-culture followed by algal re-isolation offers a practical approach to examine the persistence of algal-culture bacterial communities under mixing and may help identify candidate bacterial partners with potentially strong host-specific interactions without requiring axenic hosts.

**Supplementary Information:**

The online version contains supplementary material available at https://doi.org/10.1007/s00248-026-02840-0.

## Introduction

 The phycosphere is a microscale region of microbial interaction surrounding an algal cell [1, 2]. Within this niche, gradients in dissolved organic carbon, oxygen, and nutrients structure bacterial assemblages that influence algal growth and physiology in both engineered and natural systems [2–5]. While many of these insights were established in laboratory systems e.g., Croft, Lawrence [4], Tarakhovskaya, Maslov [5], bacterial communities in algal cultures may diverge from those of natural phycospheres because strain isolation alters the initial community and long-term culture maintenance can drive compositional shifts and reduced diversity [6–9]. These processes reflect historical contingency, where community composition is influenced by past events such as the order and timing of species immigration (priority effects) and the cultivation history of each algal strain, including its original isolation environment and subsequent laboratory cultivation conditions and media [10, 11].

Studies of phycosphere community composition and assembly typically follow two complementary approaches: community profiling and common-garden recruitment experiments. Bacterial community profiling of algal cultures examines how community composition varies with algal strain, culture conditions, and maintenance history [8, 9, 12, 13]. In contrast, common-garden recruitment experiments expose axenic algal cultures to a shared bacterial inoculum to isolate host-specific effects on community assembly [14, 15].

Across taxonomic community profiling studies, algal cultures frequently exhibit culture-specific bacterial communities that may reflect both host identity and culture history [8, 9, 13, 16]. For example, Sapp, Schwaderer [13] observed culture-specific bacterial community compositions among newly isolated marine diatom and dinoflagellate cultures. Similarly, Kimbrel, Samo [8] reported distinct bacterial assemblages associated with laboratory cultures of the marine algae *Phaeodactylum tricornutum* and *Nannochloropsis salina*, while Steinrücken, Jackson [9] identified both host and culture-medium effects across diverse algal strains. However, these studies alone cannot distinguish host selection from historical contingency and therefore risk overinterpreting strain-specific community patterns.

Common-garden experiments provide a more direct test of host-specific recruitment [14, 15]. Jackrel, Yang [14] exposed six axenic freshwater green algal cultures to a shared lake-water bacterial inoculum and detected a modest but significant phylogenetic signal, with algal phylogenetic relatedness correlating with ASV-level Bray–Curtis similarity in the recruited bacterial communities. Roager, Kempen Paul [15] compared three initially axenic marine microalgal strains from different phyla exposed to a shared seawater inoculum and found distinct host-specific microbiomes, with shotgun metagenomics revealing differences in functional potential among the recruited communities. Taken together, these studies demonstrate non-random community assembly consistent with algal host filtering. However, common-garden experiments require axenic algal cultures, which are not achievable for many strains, and evidence for a phylogenetic signal in the literature remains limited.

Most studies of phycosphere community assembly have focused on unicellular microalgae, particularly marine phytoplankton and laboratory model taxa [2, 8, 17]. Filamentous freshwater chlorophytes such as *Stigeoclonium* and *Oedogonium* are comparatively less represented in this literature, although filamentous algae are recognized components of freshwater periphyton and have biotechnological potential in wastewater bioremediation and biomass production [18].

Here, we combine two complementary designs to investigate how algal taxonomy and culture history shape bacterial community composition in long-maintained filamentous chlorophytes without the use of axenic cultures. Our focus on genera *Stigeoclonium* and *Oedogonium* was motivated by earlier work demonstrating application of a co-culture of *Stigeoclonium* sp. (K-0018) and *Oedogonium vaucheri* (K-0094) in wastewater remediation [19, 20]. These earlier studies identified markedly different bacterial communities between algal strains during monoculture, but did not investigate whether these differences reflected broader host-related patterns or the unique cultivation history of those cultures. First, a comparative strain panel from two culture collections tests whether bacterial communities cluster more strongly by algal order or by culture collection of origin, with communities profiled both in their respective long-term maintenance culture media and after transfer to a common medium. Second, a co-culture and re-isolation experiment brings two non-axenic algal cultures into direct contact and tracks bacterial community members after mixing, providing a practical test of host-specific effects on bacterial community assembly in non-axenic algal cultures. Together, these designs investigate whether patterns in bacterial community composition of long-maintained algal cultures reflect a phylogenetic signal, with closely related hosts harboring more similar communities than expected by chance, or whether strain-specific assemblages instead emerge from historical contingency and stochastic events.

## Materials and Methods

### Experiment 1: Multi-strain Culture Panel

The first experiment characterized the bacterial communities of 14 algal strains obtained from two culture collections: the Norwegian Culture Collection of Algae (NORCCA) and the Culture Collection of Algae and Protozoa (CCAP). These strains span three orders, Chaetophorales (*n* = 7), Oedogoniales (*n* = 6), and Zygnematales (*n* = 1), and had been maintained for years to decades in seven different maintenance culture media (Table 1). Strain selection was guided in part by prior work on the strains *Stigeoclonium* sp. (K-0018) and *Oedogonium vaucheri* (K-0094), and additional available related strains were included so that we could test whether cultures of more closely related algae harbor more similar bacterial communities than cultures of more distantly related algae. The single *Fritschiella* strain was included because the genus *Stigeoclonium* is polyphyletic, with described lineages distributed across Fritschiellaceae and Chaetophoraceae [21]. Formal comparisons were conducted at the order level because this provided a clear categorical factor separating more closely related and more distantly related algal strains. The single *Zygnema* strain was included for exploratory context only, representing a filamentous green alga from Zygnematales, an order distinct from Chaetophorales and Oedogoniales. Maintenance conditions at NORCCA and CCAP are similar: 15 °C, low light intensity (~ 5–10 µmol m⁻² s⁻¹), and photoperiods of 16:8 and 12:12 h light: dark, respectively. All algal strains used in this study were provided as non-axenic by the respective culture collections. While prior treatments such as antibiotic exposure cannot be excluded, no records of such treatments were available.

**Table 1:** Algal strains with maintenance media and isolation details

Collection	Strain	Order	Species	Maintenance Media	Isolation
CCAP	428/2	Chaetophorales	*Fritschiella tuberosa*	3 N-BBM + V (agar)	Allahabad, India, 1971
CCAP	477/11a	Chaetophorales	*Stigeoclonium tenue*	MWC	New Forest, England, < 1963
CCAP	477/11b	Chaetophorales	*Stigeoclonium tenue*	MWC	Derbyshire, England, < 1963
CCAP	477/13	Chaetophorales	*Stigeoclonium variabile*	MWC	Dinas Powis, Wales, < 1976
CCAP	574/1	Oedogoniales	*Oedocladium cirratum*	3 N-BBM + V	Texas, USA, 1964
CCAP	575/1a	Oedogoniales	*Oedogonium cardiacum*	3 N-BBM + V	England, UK, 1949
CCAP	575/1b	Oedogoniales	*Oedogonium cardiacum*	3 N-BBM + V	England, UK, 1949
NORCCA	K-0018	Chaetophorales	*Stigeoclonium* sp.	20%Z8 + V+ soil extract	Avernakø, Denmark, 1992
NORCCA	K-1031	Chaetophorales	*Stigeoclonium* sp.	SC	Unknown, < 2017
NORCCA	K-1032	Chaetophorales	*Stigeoclonium* sp.	SC	Unknown, < 2017
NORCCA	K-0094	Oedogoniales	*Oedogonium vaucheri*	20%Z8 + V+soil extract	Unknown, < 2017
NORCCA	K-1001	Oedogoniales	*Oedogonium cardiacum*	SC	Dry Drayton, England, < 2017
NORCCA	K-1002	Oedogoniales	*Oedogonium cardiacum*	SC	Dry Drayton, England, < 2017
NORCCA	K-1453	Zygnematales	*Zygnema* sp.	DY-V	Samsø, Denmark, 2009

*CCAP* = Culture Collection of Algae and Protozoa, *NORCCA *= Norwegian Culture Collection of Algae. Media details can be obtained from culture collection websites

Upon arrival, all strains were placed under standardized experimental culture conditions to promote growth (20 °C; full-spectrum white LED lighting at ~ 20 µmol m⁻² s⁻¹; 16:8 h light: dark) for 1–2 weeks before initial sampling in their respective maintenance media (Table 1). Strains were then transferred to a common medium (Cell-Hi-HP, 0.5 g L⁻¹; Varicon Aqua Solutions, UK) for ~ 6 weeks, with a ~ 90% volume exchange after approximately 3 weeks. Cell-Hi-HP is a commercially available medium used for industrial cultivation of a wide range of freshwater microalgae and has been used to cultivate *Stigeoclonium* and *Oedogonium* in previous studies [19, 20]. It was chosen for this study because it was previously demonstrated as suitable for these genera and it was not the maintenance medium for any of the strains.

### Experiment 2: Algal Co-culture and Re-isolation

The second experiment was a co-culture and re-isolation design with *Stigeoclonium* sp. (K-0018) and *Oedogonium vaucheri* (K-0094). The cultures used here differed from those of the same strains in Experiment 1. For Experiment 2, we used monoalgal stock cultures that had been maintained in Cell-Hi-HP medium for more than two years and had been used in previous studies that in part motivated this work [19]. Experiment 2 was initially conceived as a follow-up to this earlier work with the aim of investigating how the bacterial communities of the two algal cultures mixed during algal co-culture. Experimental culture conditions were as described above (Section Experiment 1: Multi-strain Culture Panel). The aim of this experiment was to test whether algal identity remained detectable in bacterial community composition after two algal strains were grown in co-culture and then separated.

Bacterial communities were sampled across three treatments: Stock (monoalgal cultures maintained in HP medium for two years), Washed (single filaments isolated directly from each Stock culture by capillary and regrown), and Co-cultured (post co-culture re-isolates). For the Co-cultured treatment, the two Stock cultures were co-inoculated in equal volumes into fresh medium and maintained together for six weeks, with a ~ 10% transfer (10% of culture volume inoculated into fresh medium) at week 3. Following co-culture, single filaments were washed and re-isolated by capillary to re-establish monoalgal cultures. Stock samples represent reference samples prior to experimental manipulation and were included for context rather than as replicated experimental treatments. Capillary isolation may favor tightly attached or closely associated bacteria, therefore the same procedure was applied to both Washed and Co-cultured treatments to control for bias introduced by manipulation. This design enables comparison between the Washed and Co-cultured treatments.

**Fig. 1:**
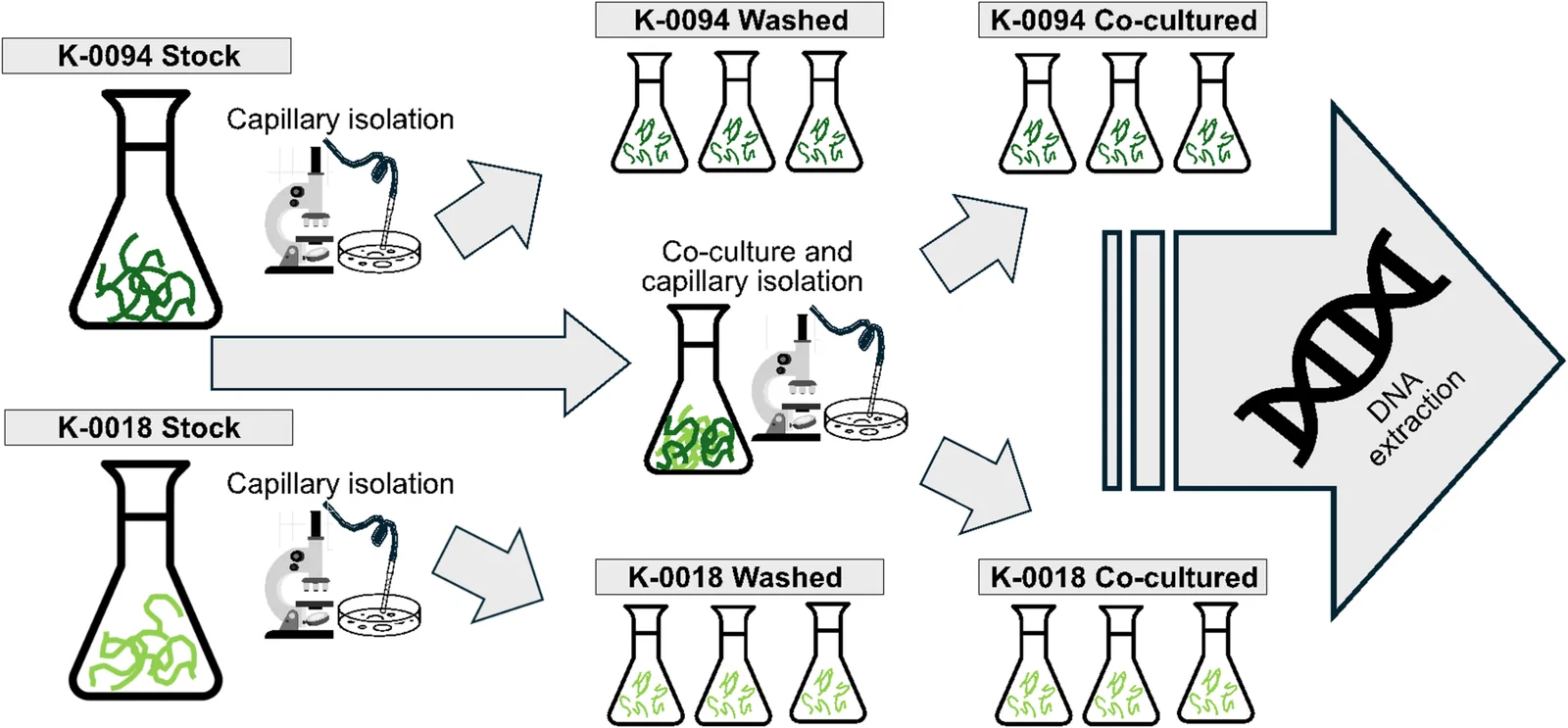
Algal co-culture and re-isolation workflow for Experiment 2. Two filamentous algae, K-0018 (*Stigeoclonium* sp.) and K-0094 (*Oedogonium vaucheri*), were processed in three treatment groups: Stock (original culture), Washed (single-filament washing and regrowth baseline), and Co-cultured (strains mixed, then re-isolated by capillary and regrown in monoalgal culture). DNA was extracted for 16 S rRNA gene metabarcoding

Manipulations were performed under a stereo microscope with sterilized tools in open, non-sterile conditions due to the need to work under the stereo microscope. Washed and Co-cultured re-isolates (filaments) were first recovered in covered 300 µL microtiter wells for one month, then verified as monoalgal cultures before transfer to 25 mL filter-cap flasks for a further two months (three months total). Cultures were then harvested and processed for 16 S rRNA gene metabarcoding (Fig. 1). Replication was asymmetric due to experimental design and sample loss (Supplementary Table 1) (Stock: *n* = 1 per strain; Washed: K-0018 *n* = 5, K-0094 *n* = 5; Co-cultured: K-0018 *n* = 5, K-0094 *n* = 3).

### DNA Extraction and Library Preparation

Both experiments used the same DNA extraction, metabarcoding library preparation, high-throughput sequencing, and bioinformatics pipelines prior to statistical analysis. Algal culture samples (~ 15 mL) were collected by filtration onto 0.2 μm pore-size hydrophilic polycarbonate membranes (47 mm, Isopore™, Millipore, Sigma, USA). This whole-culture filtration approach captured bacterial communities present in the algal cultures, without separating attached or closely associated bacteria from free-living bacteria. Filters were stored at − 20 °C until processing. Filters were cut into pieces using sterile instruments on a sterile bench, and DNA was extracted from the filter pieces with the NucleoSpin™ Soil kit (Macherey-Nagel™, Germany) using lysis buffer L-1, following the manufacturer’s instructions.

Metabarcoding libraries were prepared by two-step PCR targeting bacterial DNA from the 16 S rRNA gene V3–V4 region. The first PCR used primers 341 F (5′-CCTACGGGNGGCWGCAG) and 805R (5′-GACTACHVGGGTATCTAATCC) [22], both modified with Illumina Nextera adapter overhangs [23]. PCR reactions (20 µL) contained 1× Q5 Reaction Buffer, 200 µM dNTP mix, 0.5 µM of each primer, 0.5 µL Q5 High-Fidelity DNA Polymerase (New England Biolabs, USA), and 1–10 ng template DNA, with nuclease-free water to volume. Thermocycling conditions were: initial denaturation at 98 °C for 3 min; 25 cycles of 98 °C for 30 s, annealing at 55 °C for 30 s, extension at 72 °C for 30 s; and a final extension at 72 °C for 3 min.

In the second PCR, Illumina Nextera XT dual indexes and sequencing adapters were added with 15 amplification cycles (98 °C for 10 s, 66 °C for 30 s, 72 °C for 30 s), preceded by a 3 min denaturation at 98 °C and followed by a 2 min final extension at 72 °C. Amplicons were purified with Cytiva Sera-Mag Select magnetic beads (Cytiva, USA). Indexed libraries were pooled in equimolar concentrations (~ 6 nM per sample), quantified using the Qubit dsDNA High Sensitivity Assay Kit (Thermo Fisher Scientific, USA), and quality checked by gel electrophoresis. Sequencing was performed at the Norwegian Sequencing Centre (Oslo, Norway) on an Illumina MiSeq v3 platform (2 × 300 bp paired-end). Sequencing data have been deposited in the European Nucleotide Archive under study accession PRJEB107811.

### Bioinformatics

Raw data files were processed with Cutadapt [24] to remove primer sequences, followed by analysis in R using DADA2 [25]. Reads were truncated at 260 bp (forward) and 195 bp (reverse) and low-quality reads were filtered with a maximum expected error of 2 and a minimum read length of 195 bp. Error rates for base calls were learned from the data, dereplicated reads were merged, and chimeras were removed by consensus. Amplicon sequence variants (ASVs) were assigned taxonomy using the SILVA v138.1 database [26], which returns bootstrap support at each rank. We report the deepest rank with sufficient support: species if species bootstrap ≥ 90, otherwise the deepest rank with bootstrap ≥ 80. Reads classified as chloroplasts or mitochondria were excluded.

Potential laboratory contaminants were identified and removed using the decontam package [27] by the prevalence method and negative extraction controls. Six extraction blanks were processed alongside the samples, of which five yielded usable sequencing data and were included in the decontam analysis. After decontamination, samples with fewer than 10,000 paired-end reads were excluded. The resulting ASV tables were imported into phyloseq [28] for community analysis.

### Experiment 1: Order-level Community Composition

To describe broad patterns in bacterial community composition across the 14 algal strains, ASVs were aggregated to the order level. Relative abundances (read numbers divided by the total sample reads) of each order were calculated for each sample and the top 20 orders by mean relative abundance were retained for visualization in bar plots. This aggregation was used only for construction of bar plots, and all ordination and statistical analyses described below were performed using ASV-level data. Extraction replicates from the same culture were available for some strains and were treated as technical replicates (independent DNA extractions from the same culture). This was done to mitigate the risk of sample loss and to demonstrate the consistency of metabarcoding results. These replicates were presented as separate samples in bar plots, but were merged before statistical analyses to avoid pseudoreplication.

### Experiment 1: Ordinations

Extraction replicates were merged to a single sample per strain–medium combination prior to ordination and statistical analyses using function merge_samples() in the R package phyloseq. ASVs were included in ordinations if they had ≥ 1.0% relative abundance in any sample. We quantified ASV overlap by counting ASVs unique to one strain, shared among more than one but no more than half of strains, and shared among more than half of strains. We also calculated the mean and standard deviation of the number of ASVs per strain–medium sample included in the ordination dataset.

To assess whether conclusions were robust across complementary representations of bacterial community dissimilarity, we used four commonly applied ordination frameworks capturing abundance-weighted, log-ratio compositional, and phylogeny-informed structure. Bray–Curtis dissimilarities emphasize differences in dominant community members. Aitchison distances instead represent relationships among taxa based on centered log-ratio transformation, which accounts for the compositional properties of sequencing data. Weighted and unweighted UniFrac distances additionally incorporate phylogenetic relationships among ASVs, with weighted UniFrac emphasizing differences in the abundance of phylogenetically related taxa and unweighted UniFrac emphasizing phylogenetic turnover based on presence–absence patterns [29, 30]. Bray–Curtis and UniFrac distance matrices were visualized using Principal Coordinates Analysis (PCoA), whereas centered log-ratio–transformed data were visualized using Principal Component Analysis (PCA), which is appropriate for Euclidean distances in CLR-transformed compositional space. Phylogenetic relationships among ASVs for estimating UniFrac distances were inferred by aligning representative sequences with AlignSeqs() in the R package DECIPHER [31], masking alignment columns with at least 50% gaps, and building a maximum-likelihood tree under a GTR + Γ + I model, initialized from a neighbor-joining tree and optimized with stochastic rearrangements in R using package phangorn [32].

To test whether community composition was better explained by algal order or culture collection, we ran PERMANOVA using adonis2 (999 permutations, by = “margin”) with both factors included as main effects and extracted R² values. Analyses were performed separately for strains grown in the shared Cell-Hi-HP medium and in their original maintenance media. The single Zygnematales strain (K-1453) was excluded to avoid distortion from an order represented by only one strain. Homogeneity of multivariate dispersions was assessed with betadisper and permutation tests (999 permutations) to distinguish centroid differences from differences in dispersion. Multivariate dispersions were homogeneous across all tests except for a borderline difference among algal orders for unweighted UniFrac distances in the HP medium (PERMDISP, *p* = 0.049).

### Experiment 2: ASV-level Community Composition and Richness

Community composition at the ASV level was investigated with stacked bar plots. ASVs were first filtered to those with a maximum relative abundance ≥ 0.1% in any sample. This filter retained 95 ASVs, which were used for all ordination and statistical analyses described below (Section Experiment 2: Ordination and Community Mixing). For visualization, the 30 ASVs with the highest total relative abundance across all samples were selected and displayed by strain and treatment in stacked bar plots without further renormalization.

Bacterial richness at the ASV level was estimated from unfiltered raw counts using the R package breakaway, which infers richness while accounting for unseen taxa in sparse data with uneven sample depth [33]. Estimates were computed per sample and summarized by strain–treatment group.

### Experiment 2: Ordination and Community Mixing

Beta-diversity analyses in Experiment 2 followed the same workflow as in Experiment 1, including distance calculations and ordination methods. First, ordinations across all treatments (Stock, Washed, and Co-cultured) investigated whether the bacterial communities associated with the two algal strains converged during co-culture. Second, ordinations restricted to Co-cultured samples investigated whether algal strain identity remained detectable after re-isolation.

For ordinations restricted to Co-cultured samples, the association between ASV composition and host strain identity was tested using the function envfit() in the R package vegan (999 permutations) [34, 35]. For each ordination, we computed the Pearson correlation (r) between CLR-transformed ASV abundances and the fitted strain-separating vector and plotted the 15 ASVs with the largest |r|.

### Experiment 2: Differential Abundance

Differential abundance between the two strains after co-culture was estimated using ALDEx2 applied to raw count data [36]. Prior to modelling, ASVs were filtered using two criteria: (1) presence in at least two Co-cultured samples, and (2) a mean relative abundance of at least 0.1% across Co-cultured samples, calculated from library-size–normalized counts. Raw counts for the remaining 43 ASVs were then passed to ALDEx2, which models sampling variation using a Dirichlet–multinomial framework and performs a centered log-ratio (CLR) transformation across 128 Monte Carlo instances. Group differences were evaluated using Welch’s t-tests and standardized effect sizes with 95% confidence intervals. Given the limited replication, we highlight ASVs with |effect size| > 1 and confidence intervals that do not cross zero [36].

To test whether enrichment after co-culture depended on prior association with the host, we examined whether ASV enrichment in Co-cultured samples was predicted by presence in the Washed or Stock treatments of the same algal strain. Presence was defined as detection with > 10 reads in at least 50% of samples (Washed + Stock) within each algal host strain. For each strain, we then fitted a logistic regression model with a binomial error distribution to estimate the odds of enrichment after co-culture as a function of prior presence using glm in R, with separate models for *Stigeoclonium* (K-0018) and *Oedogonium* (K-0094).

## Results

After decontamination and removal of low-count samples, the read depth across both experiments (excluding negative controls) was: mean 26,582, median 27,486, and range 10,774 to 36,465. In total, 40 of 44 samples from Experiment 1, and 20 of 23 from Experiment 2 passed sequencing with merged paired-end read counts above 10,000 (Supplementary Table 1). All non-negative samples excluded for low reads had fewer than 1000 reads. The R package decontam identified 11 of 527 ASVs as contaminants, which were removed from the sequence table, leaving 516 ASVs in the final dataset.

### Experiment 1: Community Composition

**Fig. 2:**
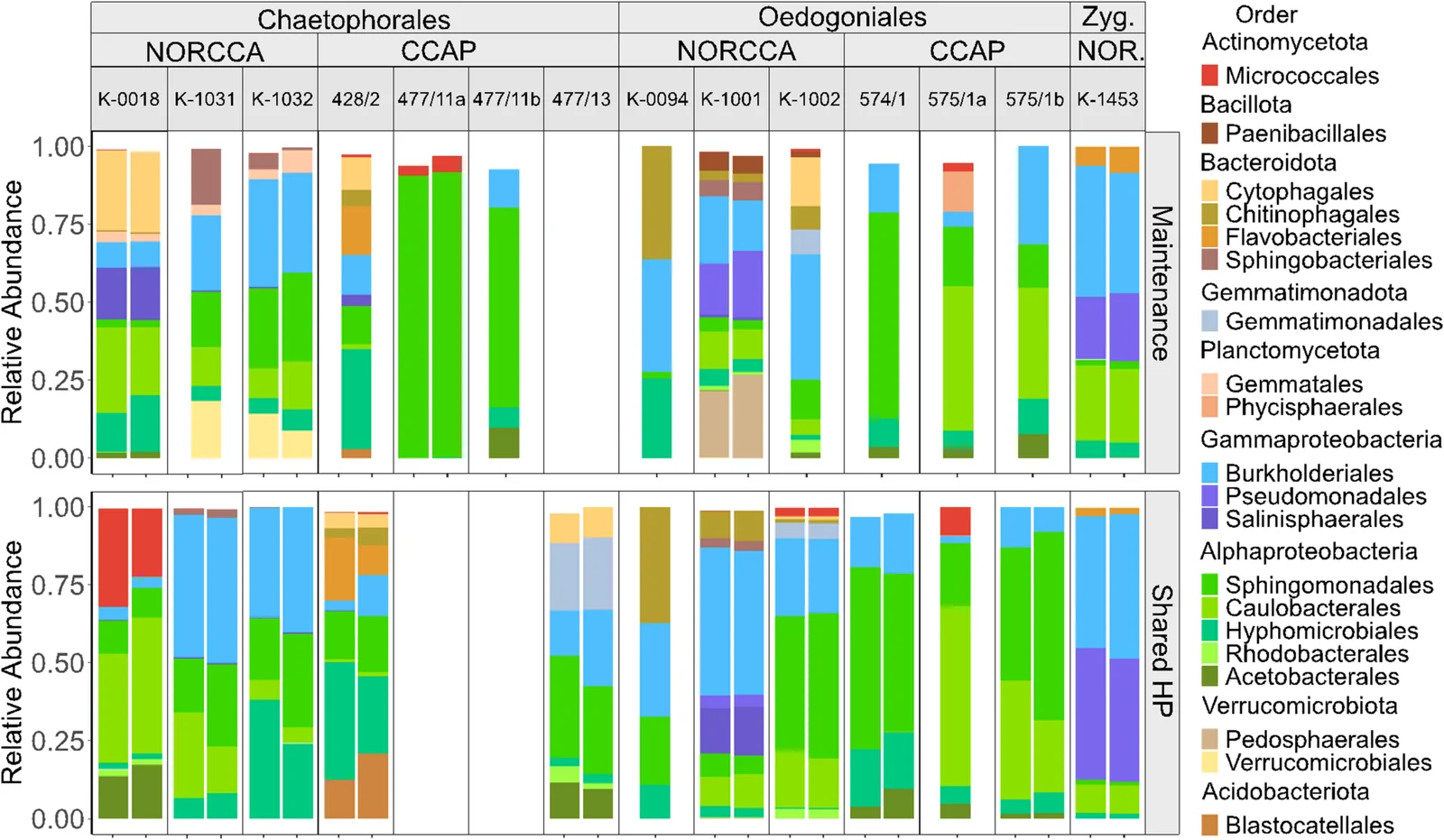
Relative abundance of bacterial orders in algal cultures. Bar plots show the 20 most abundant bacterial orders across 14 algal strains, grouped by algal order and split by collection (NORCCA, CCAP). Technical extraction replicates are shown separately. Rows distinguish cultures profiled in collection maintenance media (top) versus after transfer to a shared Cell-Hi-HP medium (bottom). Relative abundances are within sample; orders outside the top 20 are omitted (no renormalization)

Community composition of the 20 most abundant bacterial orders is shown in Fig. 2; together these orders accounted for more than 95% of total reads. Of the 14 algal strains, 11 were successfully sequenced in both media (see Supplementary Table 1), and technical extraction replicates were consistent. Communities were broadly stable after transfer to Cell-Hi-HP, although some strains showed compositional changes, such as the appearance of Micrococcales (Actinobacteriota) in strain K-0018 or the loss of Verrucomicrobiales (Verrucomicrobiota) from strain K-1031. Most cultures were dominated by a small set of orders consistently detected across strains. Burkholderiales (Gammaproteobacteria) was widespread, occurring in 12 of 13 strains in maintenance medium and in all strains sequenced in Cell-Hi-HP; strain 477/11a, the only culture lacking detectable Burkholderiales in its maintenance medium, did not yield a Cell-Hi-HP sample. Other orders within Pseudomonadota (class Gammaproteobacteria and Alphaproteobacteria), such as Sphingomonadales, Caulobacterales, and Hyphomicrobiales, were also broadly prevalent, while orders from other phyla were less consistent but still contributed substantially in several algal strains.

### Experiment 1: Ordination

**Fig. 3:**
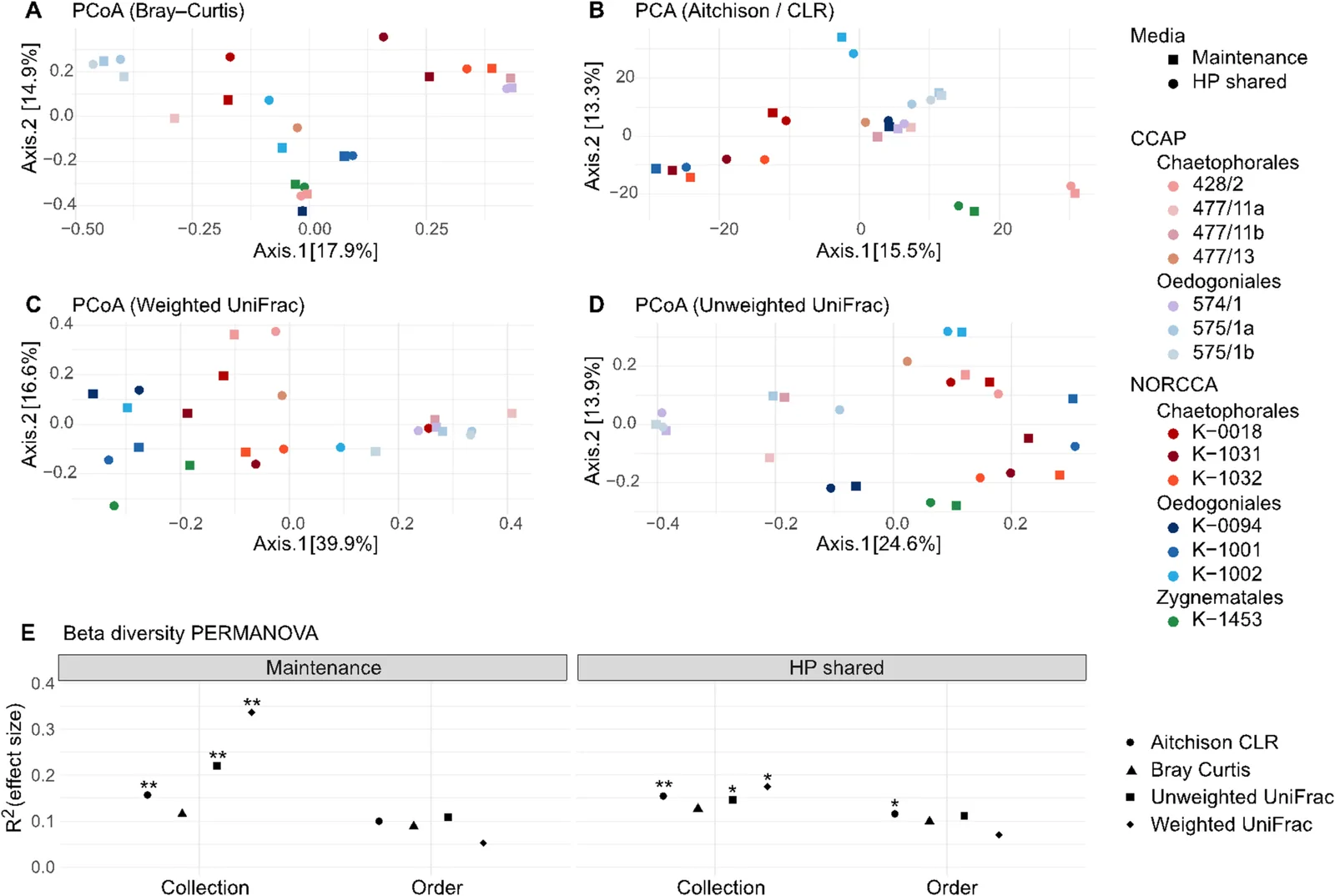
Ordination and PERMANOVA of bacterial communities in long-term algal cultures. PCoA based on Bray–Curtis (**A**), PCA Aitchison (**B**), PCoA weighted UniFrac (**C**), and PCoA unweighted UniFrac (**D**). Points represent strains grown in maintenance media (squares) or shared Cell-Hi-HP medium (circles), colored by strain. (**E**) R² values from PERMANOVA (adonis2, 999 permutations, by = “margin”) for algal order and culture collection across the corresponding distance matrices with strain K-1453 from order Zygnematales excluded. Asterisks indicate permutation p values (* *p* < 0.10; ** *p* < 0.05)

ASV-level overlap in Experiment 1 was low. After applying the 0.1% relative abundance filter, samples contained a mean of 27 ± 12 ASVs. Of the 245 ASVs retained for ordination, 157 (64.1%) were unique to a single strain, 85 (34.7%) were shared among more than one but no more than half of strains, and only 3 (1.2%) occurred in more than half of algal strains. Given this limited overlap, phylogeny-aware UniFrac distance metrics were particularly informative. Strains from different algal orders were intermixed in all ordinations (Fig. 3A–D). Chaetophorales and Oedogoniales did not cluster separately, and strain K-1453 (Zygnematales) did not separate from Chaetophorales or Oedogoniales strains despite its phylogenetic distance.

Culture collection consistently explained more variance in community composition than algal order (Fig. 3E). This pattern was strongest when strains remained in their maintenance media, where weighted and unweighted UniFrac showed larger effect sizes and permutation p values < 0.05 for culture collection. After transfer to the shared Cell-Hi-HP medium, these effects were reduced. Algal order showed smaller effect sizes and was not significant in any ordination under either media condition. Small but significant effects of collection were also detected in the Aitchison PCoA for both media conditions. Given the limited ASV overlap across strains, effects observed in non-phylogenetic distance metrics may partly reflect changes in the relative abundance of the few shared ASVs rather than broader compositional similarity.

### Experiment 2: Community Composition

**Fig. 4:**
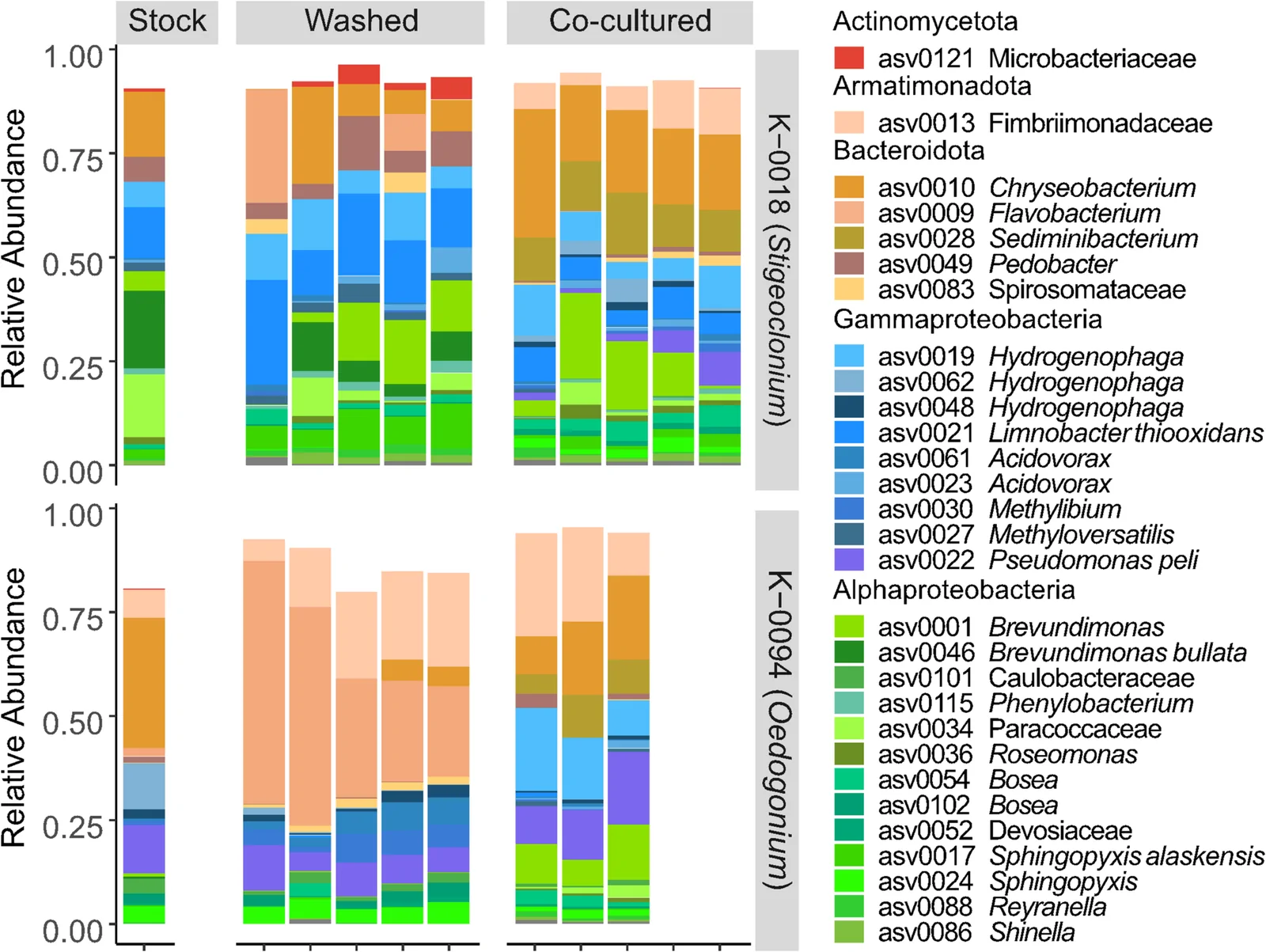
ASV-level composition and persistence of bacterial communities during co-culture. Stacked bar plots show the 30 most abundant ASVs in *Stigeoclonium* (K-0018, top) and *Oedogonium* (K-0094, bottom) across Stock, Washed, and Co-cultured treatments. Bars represent cultures. Taxonomic assignment of ASVs reflects DADA2 bootstrap-support (species ≥ 90, otherwise deepest rank ≥ 80)

Experiment 2 profiled bacterial communities associated with *Stigeoclonium* sp. (K-0018) and *Oedogonium vaucheri* (K-0094) across three treatments: Stock (original cultures), Washed (capillary-isolated monoalgal cultures), and Co-cultured (capillary re-isolated cultures after co-culture). Because the Stock treatment was represented by a single sample per strain, it was included for contextual reference only and was not used in statistical comparisons. Instead, the Washed treatment provides a baseline for within-strain variability under the same isolation and handling procedures as the Co-cultured treatment. Before co-culture, the two strains had distinct ASV-level compositions. Despite this minimal overlap at the ASV level, both strains hosted communities dominated by classes Alphaproteobacteria, Gammaproteobacteria, and Bacteroidota. Co-culturing and re-isolation resulted in substantial mixing (Fig. 4).

### Experiment 2: ASV Richness

**Fig. 5:**
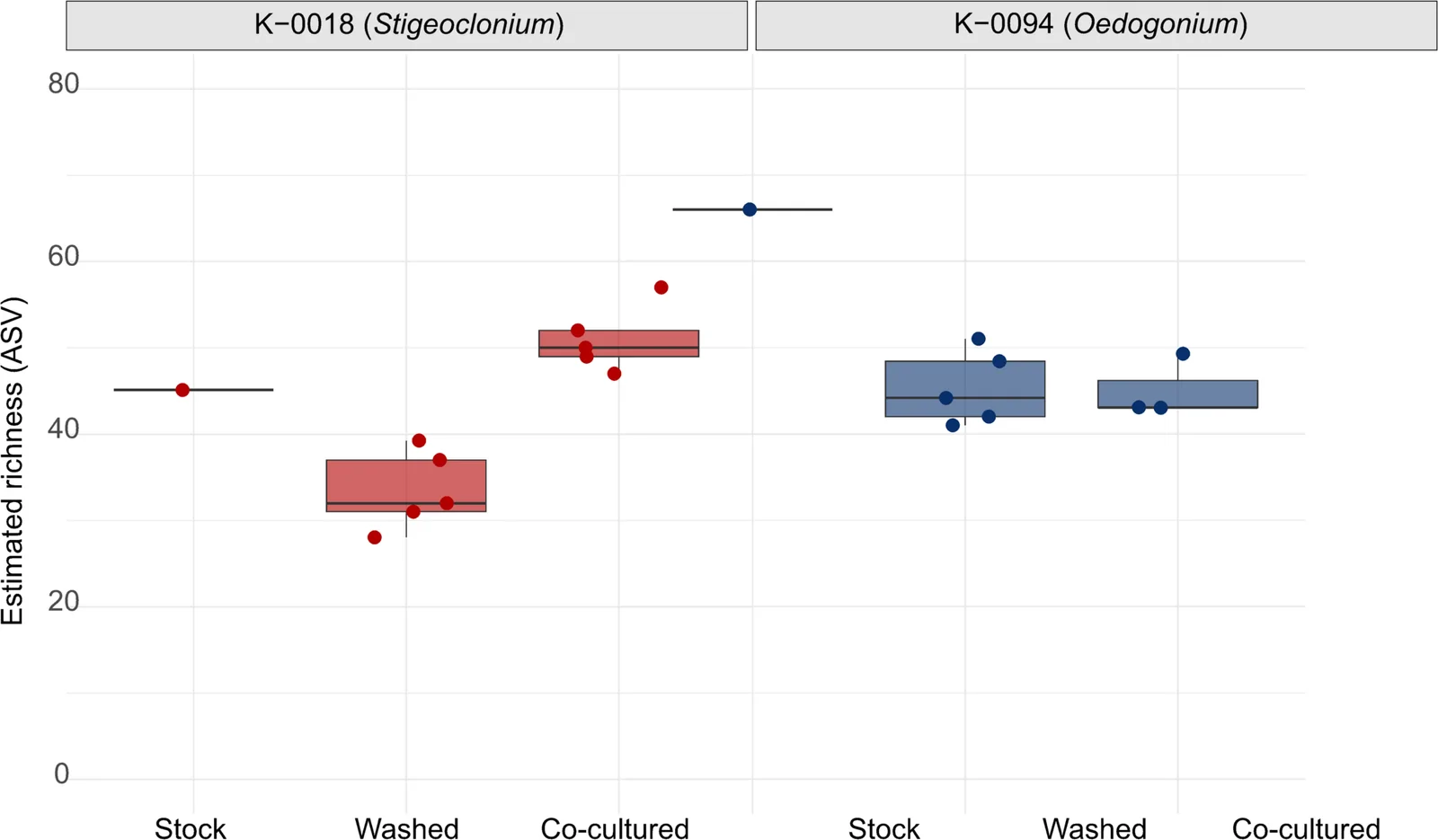
ASV richness in K-0018 and K-0094 monoalgal co-cultures. Breakaway estimates of ASV richness in Stock (*n* = 1), Washed, and Co-cultured samples. Points show richness estimates; box plots indicate median and interquartile range

In *Stigeoclonium* (K-0018), richness appeared lower in Washed monoalgal cultures relative to the Stock reference sample and then increased in the Co-cultured group. In contrast, *Oedogonium* (K-0094) showed a similar initial drop from Stock to Washed, but richness remained stable after co-culture. Because the Stock treatment is represented by a single sample per strain, comparisons involving Stock should be interpreted with caution. The primary comparison of interest is therefore between the Washed and Co-cultured treatments, which isolates the effect of prior exposure to a more diverse bacterial pool during co-culture (Fig. 5).

### Experiment 2: Ordination

**Fig. 6:**
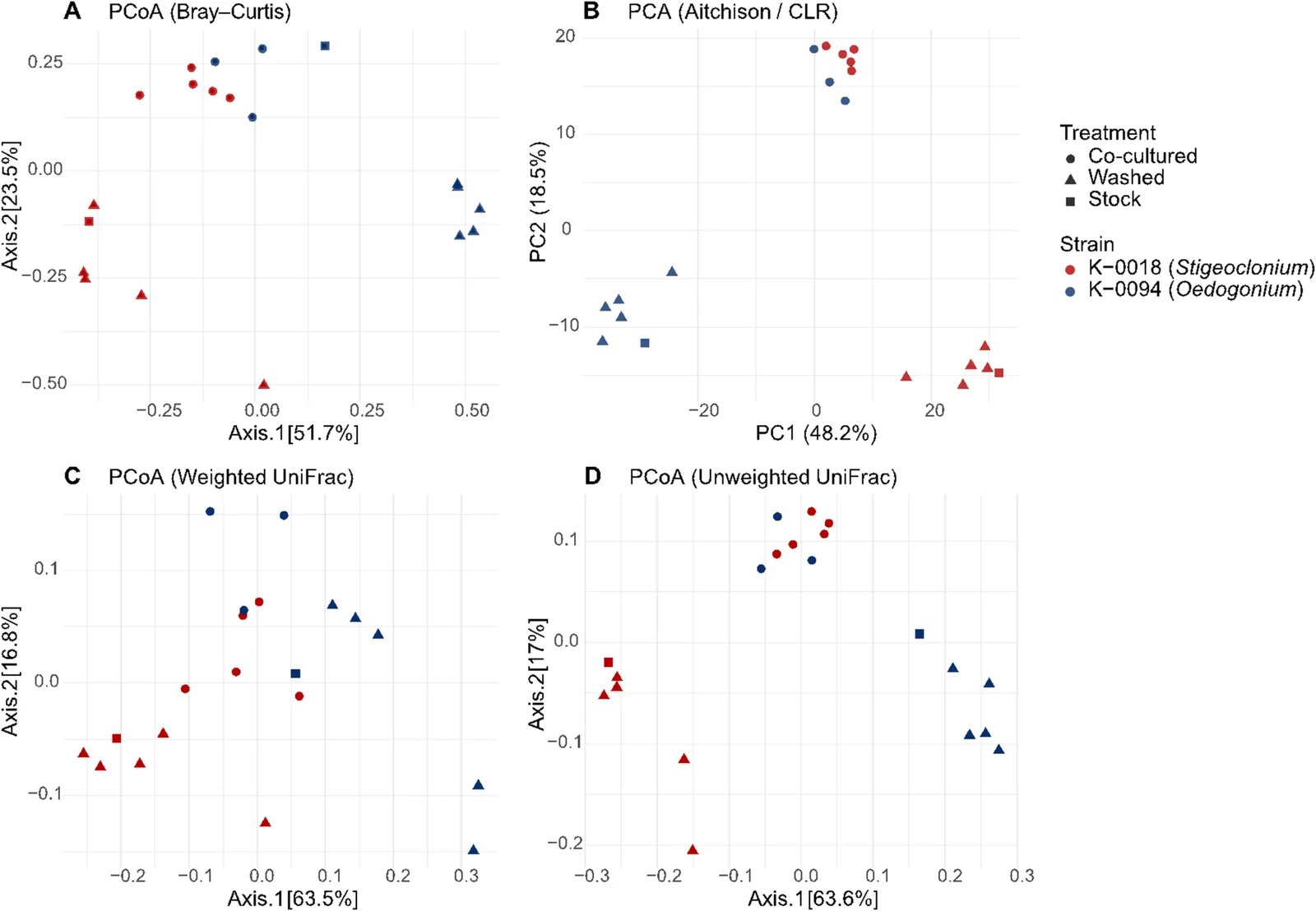
Ordination of bacterial communities in Stock, Washed, and Co-cultured samples. PCoA based on Bray–Curtis (**A**), PCA Aitchison (**B**), PCoA weighted UniFrac (**C**), and PCoA unweighted UniFrac (**D**). Shapes indicate group (Stock, Washed, Co-cultured); colors indicate algal strain. Axis labels show the percentage variance explained

Ordination of bacterial community composition across Stock, Washed, and Co-cultured groups using four distance metrics showed convergence after co-culture (Fig. 6). In general, Stock and Washed samples occupied similar regions and were distinct from the Co-cultured group in three of the four ordinations. Two exceptions were observed. First, in weighted UniFrac the separation between Co-cultured and the Stock/Washed groups was less pronounced than in unweighted UniFrac, suggesting that differences among groups were driven primarily by the presence–absence of lower-abundance ASVs, while dominant community members were more similar in abundance across treatments. Second, in the Bray–Curtis panel, the Stock sample of *Oedogonium* (K-0094) lay closer to the Co-cultured cluster than to its Washed group, a pattern not observed in the other distance metrics. This suggests greater similarity in the more abundant community members between Stock and Co-cultured samples than between Stock and Washed samples. Convergence of the two algal-culture bacterial communities was most evident in the Aitchison and unweighted UniFrac ordinations, where Co-cultured samples from both strains formed a tight cluster.

**Fig. 7:**
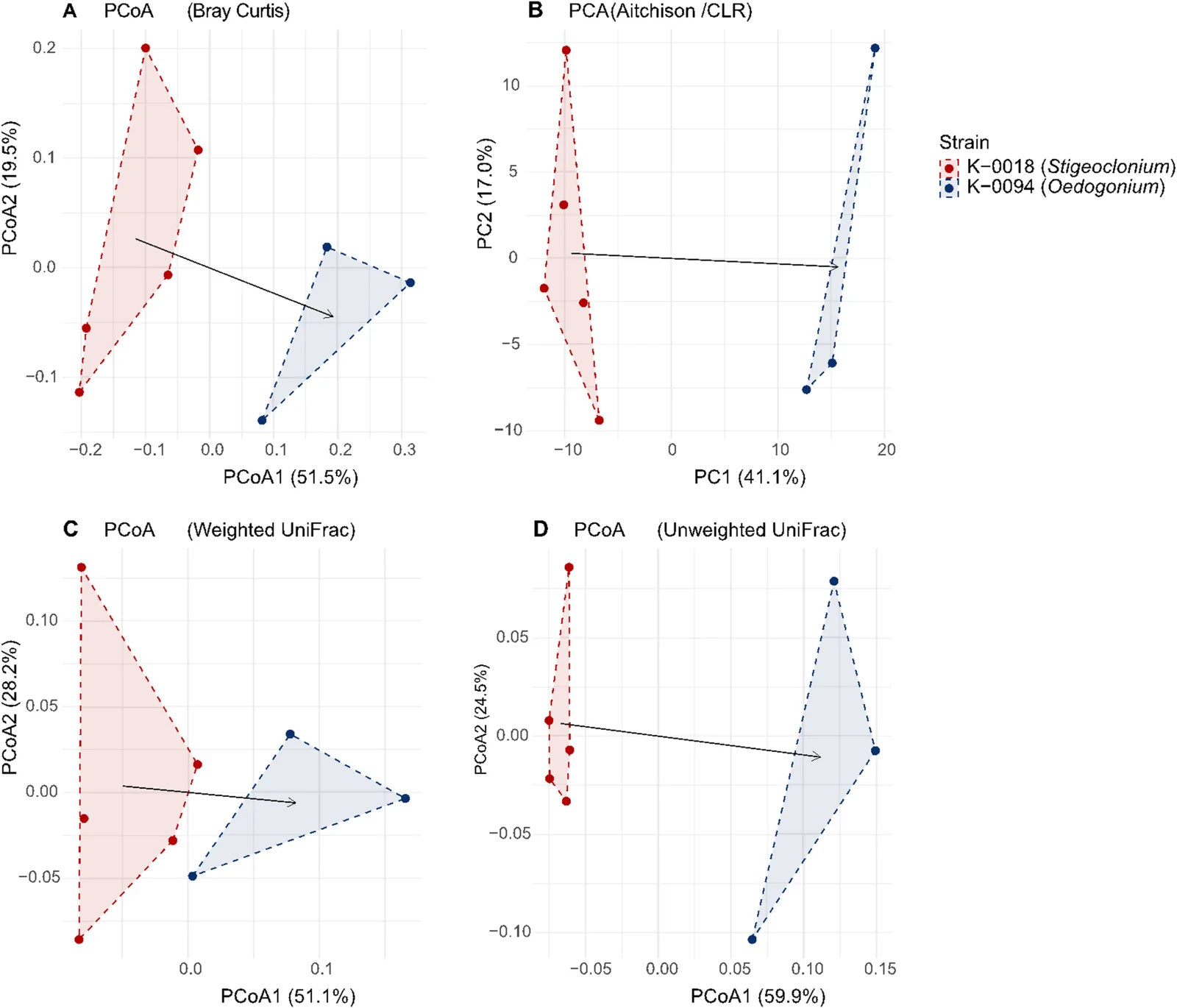
Ordination of Co-cultured bacterial communities by strain. PCoA of Bray–Curtis, Aitchison (CLR), weighted UniFrac, and unweighted UniFrac computed on co-cultured samples only. Arrows summarize how the ordination separates the two strains, as estimated with envfit(). Axis labels show the percentage variance explained

To investigate whether strain identity continued to structure communities after co-culture we re-ordinated the Co-cultured samples only, using the same four distance metrics (Fig. 7). While all four ordinations preserved some degree of strain-level clustering, the extent of separation varied across metrics. Aitchison PCA, unweighted UniFrac PCoA, and Bray–Curtis PCoA showed the clearest strain-level separation.

**Fig. 8:**
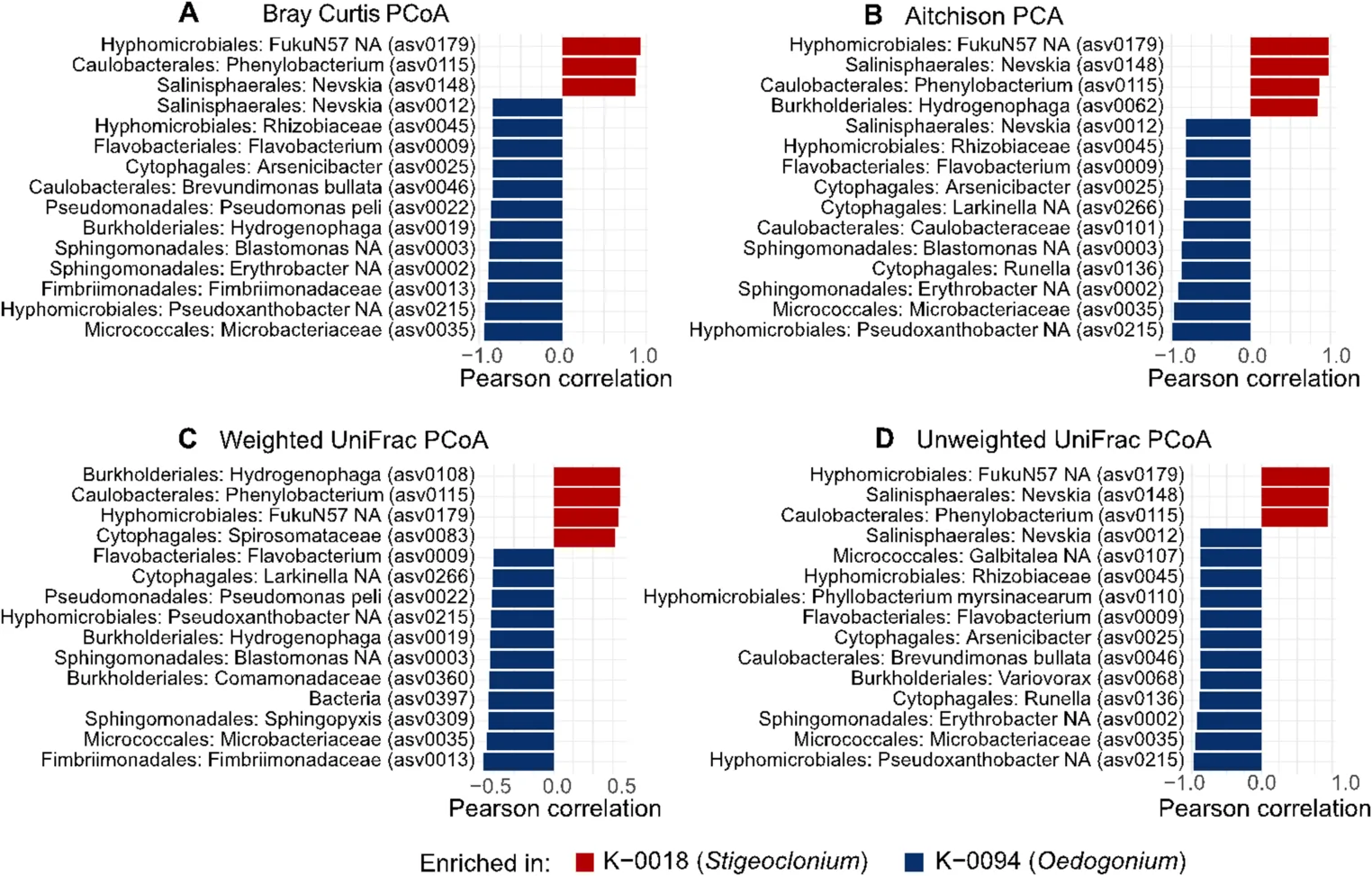
ASVs associated with strain separation in co-cultured samples. For each ordination, we computed the correlation (Pearson r) between CLR-transformed ASV abundances and the fitted strain-separating vector and plotted the 15 ASVs with the largest |r|. Positive values indicate enrichment in *Stigeoclonium* (K-0018), and negative values indicate enrichment in *Oedogonium* (K-0094). Bar color indicates strain direction (K-0018 red; K-0094 blue). Labels show the deepest supported rank per ASV: species if species bootstrap ≥ 90, otherwise the deepest rank with bootstrap ≥ 80, with order prefixed when applicable. Panels: (**A**) unweighted UniFrac PCoA; (**B**) Aitchison PCA; (**C**) weighted UniFrac PCoA; (**D**) Bray–Curtis PCoA

To identify the ASVs driving separation between strains after co-culturing, we correlated centered log-ratio (CLR) abundances with the fitted envfit vectors from each ordination (Fig. 8). Several ASVs showed strong and consistent correlations across panels. ASVs consistently correlated with strain K-0018 include asv0115 (Caulobacterales, Pseudomonadota), asv0148 (Salinisphaerales, Pseudomonadota), and asv0179 (Hyphomicrobiales, Pseudomonadota), whereas asv0012 (Salinisphaerales, Pseudomonadota), asv0009 (Flavobacteriales, Bacteroidota), asv0035 (Micrococcales, Actinobacteriota), and asv0002 (Sphingomonadales, Pseudomonadota) were consistently correlated with strain K-0094. Correlation direction did not correspond with taxonomic assignment; for example, asv0148 and asv0012, both assigned to *Nevskia* (Salinisphaerales, Pseudomonadota), showed opposite strain associations.

### Experiment 2: Differential Abundance

**Fig. 9:**
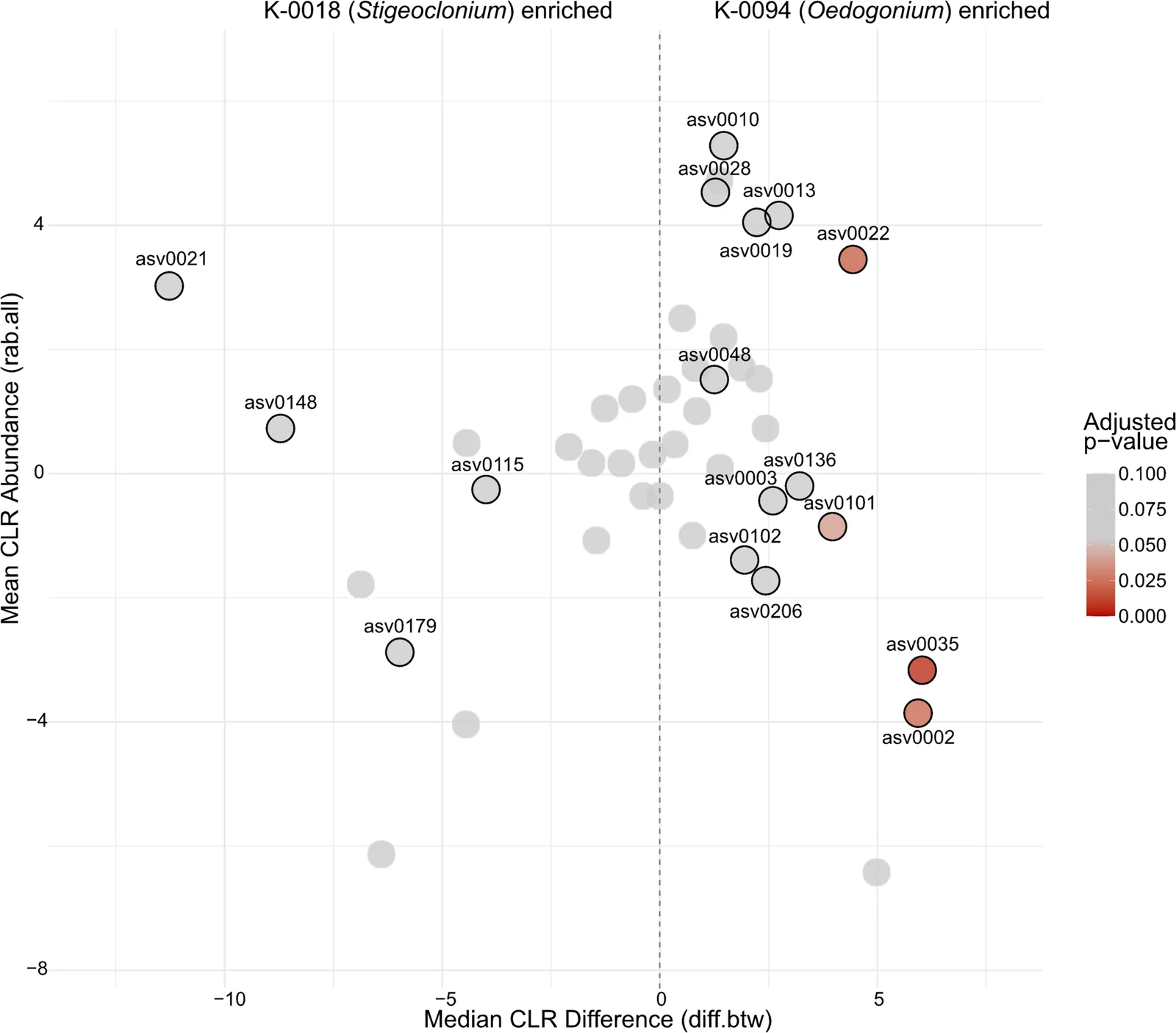
Differential abundance of bacterial ASVs after algal co-culture. ALDEx2 results comparing *Stigeoclonium* (K-0018) and *Oedogonium* (K-0094). Each point represents an ASV, plotted by mean CLR abundance (x-axis) and median CLR difference between strains (y-axis). Color indicates Benjamini–Hochberg adjusted p-values. Outlined and labelled ASVs exceed the effect size threshold (|Median CLR Difference| > 1) and have a 95% confidence interval that does not cross zero. The dashed line marks zero difference

Differential abundance analysis (ALDEx2) indicated that most ASVs had effect sizes (Median CLR Difference) near zero, consistent with broadly similar relative abundances between *Stigeoclonium* (K-0018) and *Oedogonium* (K-0094), while a subset showed clear directional shifts (Fig. 9). Given limited replication, we report adjusted p values but emphasize features with |effect size| > 1 and 95% CIs that do not cross zero [36]. Under this rule, enrichment was more common in K-0094 and included ASVs also highlighted by Pearson correlations with the envfit strain vector (Fig. 8), notably asv0002 (Sphingomonadales, Pseudomonadota), asv0035 (Micrococcales, Actinobacteriota), and asv0022 (Pseudomonadales, Pseudomonadota). Fewer ASVs were enriched in K-0018; however, several corresponded with correlations from the ordination analysis, including asv0148 (Salinisphaerales, Pseudomonadota), asv0115 (Caulobacterales, Pseudomonadota), and asv0179 (Hyphomicrobiales, Pseudomonadota). One exception was asv0021 (Burkholderiales, Pseudomonadota), which was not highlighted by the correlation approach but showed the largest effect size in ALDEx2 and high relative abundance in K-0018, although its adjusted p value was high. Together, these results indicate that co-culture did not fully homogenize the bacterial communities and that strain identity remained detectable at the ASV level.

To test whether enrichment in the Co-cultured samples could be predicted by prior ASV presence in non-co-cultured samples, we fitted logistic regression models for each strain. In both cases, ASVs present in either Washed or Stock cultures had odds ratios greater than 1 for enrichment after re-isolation (K-0018: OR = 1.22, 95% CI = 0.20–7.53, *p* = 0.832; K-0094: OR = 1.38, 95% CI = 0.41–4.62, *p* = 0.607). However, confidence intervals were wide and included 1, indicating that these effects were not statistically significant. Prior presence in long-term laboratory cultures was therefore at best a weak predictor of enrichment after co-culture and re-isolation.

## Discussion

Although the strain panel (Experiment 1) and the co-culture and re-isolation experiment (Experiment 2) differed in scope and design, they converged on the same pattern: bacterial communities in algal cultures shared a broadly similar composition at higher taxonomic ranks but were culture-specific at the ASV level. These ASV-level differences are consistent with historical contingency and culture history rather than algal taxonomy, although within this stochastic background algal hosts may still maintain or favor a limited subset of strain-specific bacterial partners. Experiment 1 showed that culture-specific bacterial communities were not predicted by algal order. Experiment 2, in which strains shared an environment, showed that while communities largely converged when allowed to mix, a small subset of ASVs consistently differed in relative abundance after co-culture and re-isolation. Because DNA was extracted from whole-community filters, the analysis includes both algae-associated and free-living bacteria. However, in these small-volume laboratory cultures with high algal biomass, where organic carbon is algal-derived, community composition is expected to be dominated by algae-associated taxa. Because all treatments were processed consistently, this does not affect the relative comparisons among treatments.

### Culture History Outweighs Algal Order

Across algal strains we observed a shared higher taxonomic structure, consistent with prior reports [8, 9, 13, 17]. In both marine and freshwater systems, phycosphere communities are frequently dominated by Bacteroidota and Pseudomonadota, phyla that include many taxa with functions thought to promote algal–bacterial associations, such as vitamin exchange, chemotaxis, and degradation of algal substrates [2, 3, 37]. However, the species that fill these roles differ among algal cultures and between studies [2, 3, 37, 38]. Such variation is consistent with host-specific associations acting within conserved functional guilds, but it can also arise from historical and stochastic processes, including founder effects, arrival order, and laboratory maintenance conditions [10, 11, 39].

The results of Experiment 1 suggest that bacterial communities of laboratory cultures of Chaetophorales are not more similar to each other than those of Oedogoniales cultures. While this result does not exclude the possibility of host filtering, it supports the role of stochastic processes in structuring the bacterial communities of filamentous chlorophyte cultures. Culture collection was a stronger predictor of community structure than algal order, suggesting that historical contingency and long-term maintenance environment shaped bacterial composition more than host order-level taxonomy. Phylogeny-aware UniFrac distances were particularly informative given the low bacterial ASV overlap among strains and showed the largest collection effect under maintenance media conditions, suggesting that strains sourced from the same culture collection hosted more phylogenetically similar bacterial communities.

Because culture collection and maintenance medium were confounded, we standardized culture media and conditions, which reduced but did not eliminate these differences. Standardizing the media controlled for differences in the chemical environment of the cultures but not for the historical effects, which we therefore consider part of culture history. We do not attempt to disentangle the effects of culture collection from those of historical maintenance media but instead highlight that culture history continues to imprint structure on the bacterial communities of algal cultures, and that this effect exceeds that of order-level taxonomy within the tested set of cultures.

A similar pattern was qualitatively apparent between the K-0018 and K-0094 cultures used in Experiments 1 and 2, which differed in bacterial community composition despite representing the same algal strains. Although not formally tested here, this observation is consistent with the strong influence of algal-culture history on associated bacterial communities. The present strain panel also represents a limited phylogenetic range within filamentous freshwater chlorophytes, and therefore the absence of a detectable order-level effect should not be interpreted as evidence that algal phylogeny is generally unimportant for bacterial community assembly across broader algal diversity.

### Partial Convergence of Bacterial Communities

Experiment 2 tested whether algal strains maintained different bacterial communities when cultures were allowed to mix and subsequently separated. This exposed both algal strains to a shared bacterial pool composed of the combined bacterial communities of the two algal strains. Under these conditions, the two algal-associated bacterial communities partially converged as bacterial partners moved between hosts. However, a subset of ASVs remained consistently enriched in one algal strain after co-culture, indicating that host identity can maintain some differences when strains have access to the same bacterial pool. This pattern was supported by both ordination and differential abundance analyses and, although limited by low sample number, suggests a real biological effect.

Similar patterns have been reported in common-garden recruitment-based studies where diverse algae are exposed to shared environmental bacterial communities and subsequently recruit partially distinct phycosphere assemblages [14, 15]. This is further supported by evidence that algal-bacterial interactions are often host-specific, with the same bacterial isolate producing different effects across algal hosts [40]. Unlike these other axenic culture-based recruitment studies, the shared bacterial pool in the present study was already composed of taxa that had persisted during prolonged laboratory cultivation with one of the two algal hosts, meaning that the experiment tested host-associated partitioning within an already algae-adapted bacterial community rather than recruitment from a broader environmental pool.

The co-culture samples do not represent a direct measurement of communities during co-culture, as composition likely shifted during regrowth following capillary isolation. However, they provide a useful contrast, as both strains were re-established from a shared microbial pool and subsequently exhibited differential enrichment of specific ASVs. Direct sequencing of individual filaments would involve very low biomass and introduce technical challenges, but more importantly would capture only a transient mixed community rather than host filtering during regrowth. Although we did not sample across a time series and cannot confirm that a stable state was reached, the re-isolated cultures represent communities that developed under host-specific conditions following exposure to a shared bacterial pool. We therefore interpret the differential abundance results as evidence that community composition in the re-isolated monocultures following co-culture differed between the two algal strains.

Ordination-based correlations across four distance metrics, together with ALDEx2 differential abundance, highlighted many of the same ASVs as strain-associated after co-culture, which strengthens confidence that these patterns are robust. For *Oedogonium* (K-0094), asv0022 (*P. peli*, Pseudomonadales) provides a clear example: it moved between hosts during co-culture but remained more abundant in K-0094 after re-isolation. For *Stigeoclonium* (K-0018), asv0021 (*L. thiooxidans*, Burkholderiales) showed similar strain-linked enrichment. Several additional ASVs followed these patterns. However, prior presence in Stock or Washed cultures only weakly predicted which ASVs became enriched after co-culture. This mismatch suggests that many apparent strain-specific patterns in long-maintained algal cultures reflect historical contingency rather than stable host-selected associations, consistent with studies of long-maintained diatom cultures showing strong effects of culture history and persistent legacy of the isolation environment on microbiome composition [12]. In contrast, the small subset of ASVs that remained consistently strain-biased after co-culture, despite not aligning closely with prior presence, are more likely to reflect true host-linked associations. Together, these patterns suggest that bacterial communities of cultures of the two algal strains are neither fully interchangeable nor strongly host specific but instead reflect a mixture of history-driven variation with modest, reproducible host-linked differences [8].

The co-culture and re-isolation approach provides a practical framework for studying algal-associated bacterial assembly without requiring axenic algal cultures. Rather than testing recruitment from an environmental inoculum, the design examines how previously established algal-associated communities reorganize after community mixing and re-isolation. Because the approach relies on physical re-isolation rather than axenic cultivation, it may be useful in systems where establishing or maintaining axenic cultures is impractical and could potentially be extended to larger pairwise or polyculture community mixing experiments. Molecular confirmation following re-isolation could extend this approach to morphologically similar strains. Furthermore, the logistic regression approach testing whether prior presence predicts post re-isolation enrichment could similarly be expanded to more complex polyculture designs.

## Conclusion

Bacterial community composition in long-maintained algal cultures was shaped more by culture collection and maintenance environment than by algal order. Transferring algal strains to a common medium reduced but did not remove these differences, consistent with a lasting imprint of culture history. In the co-culture experiment, two initially distinct communities mixed, yet a small set of ASVs remained differentially abundant between the algal strains after re-isolation. These host-linked abundance differences suggest that algal host identity can contribute to bacterial community structure when strains share a bacterial pool. Co-culture followed by re-isolation offers a practical way to identify bacterial partners with algal-strain-specific associations without the need for axenic algal cultures. Identifying bacterial partners that show reproducible host associations may guide future functional assays that test mechanisms and direction of effect, and may ultimately support efforts to design phycosphere communities for algal biotechnology.

## Electronic Supplementary Material

Below is the link to the electronic supplementary material.

Supplementary Table S1. Sample metadata and 16S rRNA gene amplicon read counts. Columns show sample number, experiment, culture collection, algal strain, treatment, sample result, reads retained after DADA2 processing, and reads retained after contaminant removal with Decontam. Samples marked “Fail” were excluded from downstream analyses.

## Data Availability

The sequencing datasets generated and analyzed during this study are available in the European Nucleotide Archive (ENA) under study accession PRJEB107811. All other data supporting the findings of this study are available from the corresponding author upon reasonable request.
